# A framework for infants’ emerging affect-cognition interface

**DOI:** 10.1007/s12144-026-09271-7

**Published:** 2026-07-14

**Authors:** Margaret W. Sullivan, Pimjuta Nimmapirat, Nancy Fiedler

**Affiliations:** Environmental, Occupational and Health Sciences Institute, Rutgers School of Public Health, EOHSI, 170 Frelinghuysen Rd., Piscataway, NJ 08854 USA

## Abstract

This paper advocates integrating affect neuroscience network and appraisal theories of emotion in a framework for the purpose of better describing and studying the emerging links between infant affect processes and cognition. Panskepp’s empirical and theoretical work on affect networks (1998) and Scherer and Moore’s working model of affect processing (2019) provide the theoretical underpinnings of the proposed developmental interface of affect and cognition. The framework describes early affect-cognitive links as continuously unfolding processes arising from everyday commerce with people and objects that are functional by 7 months. It assumes that affect processing accompanies all infant-relevant-environment transactions, but that the experiences infants have and the actions of which they are capable, are constrained at each age by their motor development and appraisal capacities. It hypothesizes that infant brains appraise recurring contexts, remember predictions including the affect and the appraisals correlated with them, and generalize across contexts. By 9 months, appraisals of control, intention, social cognition, and self-relevance become increasingly important in determining affect experience. By two years, self-referential processes influence affect and appraisal.

## A framework for an emerging affect-cognition interface

Recent neuroscience theory suggests that infants are incapable of emotion experience prior to the emergence of categorical concepts (Barrett, 2020; Hutchinson & Barrett, 2019). Arguments against this view generally assert that emotion experience involves more than conceptual knowledge and labeling of emotion (Izard, 2009; Lazarus, 1991; Lewis & Michalson, 1983; Zajonc, 2000). It is reasonable, based on findings in language acquisition, concept learning, discrimination of facial expressions, and social refencing that by 9 months of age, emotion and cognitive processes are linked, thereby allowing experiences and conceptual knowledge about emotion to attain new levels (Hoemann et al., 2019, 2020; White et al., 2019). The question remains: what is their interface before that age and how do infants process and experience affect while their conceptual and verbal skills are still limited?

With reference to nonverbal or minimally verbal infants, consider two related points. First, equating emotion experience with verbal emotion conceptual knowledge precludes the possibility of an earlier emotion-cognition interface arising from direct experience. Statistical and basic learning processes operate from the opening months of life and might support early affect-cognition interactions (Mascolo, 2022; Plate et al., 2022). A specific, perceived ‘feeling’ may ultimately become a concept with a label, but that concept has roots in the infant’s experience of its world. Infants acquire and remember a good deal of information about the social and physical world before they label people, objects, and actions or have categories for them. It is unlikely that this information is without affect if a term other than “emotion” is preferred prior to language concepts.

Second, restricting emotion experience to conscious verbal, conceptual knowledge ignores theory and evidence about affect’s motivational and evaluative functions. Affect in this paper refers to the qualitative aspect of experience-acquired information. It varies with the intensity of arousal and the direction of action impulses. Comparative neuroscience has established that all mammals share several primary brain-based, valenced networks that enable affect (Davis & Montag, 2019; Montag & Davis, 2018; Panksepp, 1998). These neural networks are conserved across brain evolution and are the basis of motivated behavior in mammals. Some research suggests some affect-processing networks are “live” in infants. Functional near-infrared spectroscopy supports that there is a developmentally continuous organization of pre-cortical networks involving affective processes related to attention and memory in infants. Medial (primarily affect) and lateral (primarily cognitive) prefrontal cortices also are active during the first year as well as rapidly developing connections between the amygdala and the prefrontal cortex (Grossmann, 2013; Tottenham & Gabard-Durnam, 2017). Cortical and cerebellar areas associated with memory and affect also undergo explosive development between 3 and 13 months (Choe et al., 2013). Although science has limited ability to study affect-processing networks and their integration with emerging cognition in infant brains directly, it is possible to outline an emerging interface during the first two years given what is already known about infant behavior. A framework can inform when and how hypotheses about early affect-cognition links may be tested.

Many investigators who study the emotion-cognition interface in infancy tend to focus on specific tasks or processes in context to demonstrate the interplay between affect and cognition. A recent model (See Wolfe & Bell, 2023) nested the “cognition-emotion interplay” and its association with physiological functioning within an overarching model of developing self-regulation. Although this model provides a theory-driven approach to infants’ emotional development, the nature and potential interactions between specific affect networks and cognitive processes remain unspecified. In seeking to specify the developmental aspects of that “interplay”, we describe an emerging affect-cognition interface based on established findings in each of these domains. The proposed framework integrates affect neural networks theory and appraisal theory, two theoretical perspectives that are not themselves developmental, but which have under-appreciated implications for how affect may interface with cognition. First, a summary of each model follows with a discussion of its developmental implications. Next, a justification for inclusion of infant facial displays as affect signals is discussed despite theoretical controversies regarding their meaning. Finally, we describe framework itself.

## Two theoretical perspectives

### Panksepp’s affect networks

Panksepp’s neuroscience affect theory (Davis & Montag, 2019) is not a developmental theory, but it is biologically based and defines a subcortically organized, interacting set of neural networks for affect processing. Of the multiple affect neural networks studied by Panskepp, four have relevance for infants’ developing affect-cognitive interface: seeking/interest, anger, sad/distress, and fear, each marked by facial and behavioral signals of their activation (Sullivan, 2014). These affect networks are likely activated during infants’ daily commerce with the social and non-social world. They are hypothesized to be present at birth and develop rapidly thereafter. The four networks energize either approach of or withdrawal from salient elicitors or events. Most infant emotion theorists accept them as among the primary human affects. Each is associated with distinctive behavioral signals, including facial expressions in primates.

Approach networks include seeking/interest and anger. The seeking/interest network signals positive approach affect and openness to experience while anger is a *negative* approach affect (Carver & Harmon-Jones, 2009; Harmon-Jones, 2019). In the animal literature, anger has been studied as the construct “frustrative non-reward.” Here, it will be referred to as anger/approach to differentiate it from rage/fight, a high/arousal, aggressive response characterized by movement *against* not merely toward something. Both seeking/interest and anger/approach networks energize impulses directed toward obtaining or regaining goals or objects. They are mutually exclusive (i.e. cannot be activated simultaneously) but each interacts with withdrawal networks through excitatory and inhibitory neural influences (Panksepp, 2011). Fear and sad/distress are withdrawal networks. While anger and fear emotion are often conceptualized as “emergency” fight/flight responses, these networks also function under conditions of low to moderate arousal, the typical conditions under which infants display them.

Panksepp’s model asserts that complex, *organized* networks are not located in discrete brain areas but involve communication between subcortical and cortical areas. They do not give rise to discrete states or expressions but interact in response to eliciting contexts. Since brain development, especially subcortical-cortical connectivity is incomplete in early infancy, it is likely that these networks expand during infancy. While most theories acknowledge emotional development unfolds gradually during infancy, how it does and the possibility that interacting affective-cognitive processes underlie observable affect displays is seldom considered.

### Scherer’s appraisal theory

Appraisals seem essential to the brain’s predictive functions as proposed by neuroscience. They are central to adults’ affect processing and in their simplest form, can be defined as dichotomous evaluations that underlie emotion experience (Scherer, 2001; Scherer & Moors, 2019). Scherer and Moors (2019) proposed that the integration of appraisals with physiological responses and motor/motivation components (valence, approach-withdrawal) of emotion occurs early in affect processing. Affect processing in this view is the integration of physiological changes, action tendencies, and appraisals. In Scherer’s model, appraisals occur before conscious awareness of “feelings” or emotion differentiation, and prior to other higher order cognitive processes. Categorization and labeling may occur downstream as part of higher-order processing, but these later cognitions are viewed as optional processes (Scherer & Moors, 2019). Emotion may occur without categorization or labeling and need not be expressed or signaled externally. The questions for infancy are whether any appraisals are possible, when, and how they interface with other affect processes. While the development of appraisals has been relatively unstudied (Walle et al., 2022), early occurrence of primary appraisal processes is proposed in our framework.

While not explicitly developmental, Scherer’s theory proposes that one or more appraisals always occur in response to encountered salient objects, persons, or events and influence physiological and motor action impulses (Scherer, 2001; Scherer & Moors, 2019). Davis et al. (2023) also proposed that infant appraisals (1) are an important *antecedent* of emotion differentiation (that is, infants’ ability to perceive and categorize discrete emotions) and self-regulation, and (2) are continuously calibrated by caregivers’ selective and contingent responding. Similar to Scherer and Moors (2019), they proposed primary appraisals specifically related to novelty (“What is it?”), pleasantness (“Is it good?”), and goal-relevance (“What does it mean for my goals?”). These hypothesized “appraisal checks” are basic evaluations that provide evidence for a working developmental sequence of an emerging emotion-cognitive interface. Our developmental framework adopts Scherer & Moor’s proposed appraisal list to hypothesize affect-appraisal processing prior to categorical emotion concepts.

## Infant facial affect: Justification and issues set aside

Although current facial coding systems have limitations and the notion of discrete emotion states and expressions has been largely discredited (Barrett, 2020; Camras & Shutter, 2010), infant facial affect remains an important element of the proposed framework. Including facial affect in the model is necessary because it is one way that affect processing can be inferred when the infant’s behavioral repertoire is limited. Considering the continuum of facial affect theories (Barrett et al., 2019), we treat facial expressions consistent with functionalist theories and social-affect development. [See Buss et al., 2019 for brief summaries of competing theories of infant facial affect which will not be covered here.] To clarify what we mean by facial displays and justify including them in the framework, we emphasize that: (1) infants’ facial displays, defined as correlated component movements in the upper, lower, and mid-face regions, are visible signals of affect processes– a smile may not always signal happy emotion, but it does signal that a change in infant arousal and affect processing is occurring (Sroufe & Waters, 1976); (2) facial movements are part of infants’ behavioral repertoire from birth, associated with their behaviors in context, and responded to by social partners holistically as they occur during interaction; and (3) they are graded signals, with more intense arousal associated with “maximally” discriminable facial movements. “Maximal” signals approximate the modal expressions of children (Barrett, 2020; Izard, 1995; Cole & Moore, 2014) or are blends of these movements^1^; and (4) they are minimally influenced by social display rules initially.

We acknowledge that cultures may differ in how specific sets of correlated facial components are interpreted, but maximal expression displays, their associated action tendencies, and physiological changes are expected to be similar across cultures due to their common underlying subcortical and midbrain organization and the enervation of facial musculature. Socialization and social learning begin to influence them during the first year as studies of reinforcement effects on infant smiling, imitation, and social refencing have established. It is *not* assumed that all facial behavior is “wired” to specific elicitors to an emotion center in the brain or are static displays conforming to an expression template. Rather, they are one aspect of behavioral response to the immediate context perceived by infants. Besides the perceived context, arousal level, previous experience with elicitors, and infant temperament are all presumed to influence observed facial displays. Displays and action tendencies related to them are *signals* of affect processing within neural networks, not states. Immaturity and processing constraints of neural networks may mean that the patterning and sequencing of facial movements, as well as their associated action patterns, may change with maturation although there is limited data on this question.

Developmental literature has been mired for decades in debate about *when* facially expressed affect reflects specific emotion states and how to label whole face displays so as not to imply equivalence with adult expressions. This paper sets issues of emotion differentiation, specificity, and equivalence to adult emotion aside and suggests that they are not answerable without a better appreciation of the developing affect-cognition interface and the maturation of affect processing networks. A case in point is the finding of poor differentiation of negative affect. The literature suggests that anger, sad/distress, and fear facial movements do not show specificity to eliciting contexts. Modal expressions and some context specificity for positive expression occurs by four months but specificity of negative expressions has been more difficult to establish (Bennett et al., 2002, 2005; Malatesta & Haviland, 1982). Insights from affective neuroscience may help clarify why this is so. Withdrawal (sad/distress and fear), as opposed to approach affects, first dampen approach and then either help maintain distance or facilitate movement away from the eliciting context. They have negative valence but are not disorganized or undifferentiated processes. In Panksepp’s model, fear and sad/distress share excitatory neural influences while interest and anger/approach each have both excitatory and inhibitory neural influences on both withdrawal affects. Thus, complex neural interactions are possible, perhaps explaining why young infants’ facial affect is so variable and rapidly shifting as arousal levels change over time. Infants may either approach or withdraw from stimuli in any given context. They may display blended affect signals, or even vacillate between action tendencies, showing classic approach/withdrawal conflicts. Thus, blending or mixing of approach and withdrawal affect is expected and not poor emotion differentiation. When mixed or competing affect systems are activated, appraisals may become the ultimate arbiter of specificity. Input from social partners and prior experience with contexts are likely to influence infant appraisals (Davis et al., 2023).

The question of what and when infants understand about discrete emotion expressions of others is also set aside. Pre- and early conceptual understanding of emotion may involve only basic discrimination of qualitative differences in others’ discrete affect. Data on postnatal discrimination and categorization expressions may not occur for specific, discrete negative expressions until 10–18 months (Ruba & Repacholi, 2019). Social cognition studies show that infants are sensitive to contextually embedded emotions of others by 12 months (Reschke et al., 2017) and differentiate adults’ discrete emotions displays in certain naturalistic contexts by 24 months (Walle et al., 2017). Social cognition affects understanding of emotion of self and others, so its key developmental milestones are noted in our framework. However, as noted above, we consider this differentiation “downstream” from the basic affect-appraisal processing. We do not claim that early affect-cognition links reflect early discrimination of affect although their development likely ultimately contributes to emotion understanding and verbal categorizing.

## The framework

Mapping early cognitive appraisal capacity against affect processes may suggest testable hypotheses about when the interface becomes functional and allow exploration of how temperament, social experiences, context, and other factors influence its further development. The framework in Table 1, is not a complete theory, nor a review of the literature. It is not a theory of social cognition. It only outlines the parallels between affect and cognitive processes in the first two years that may index the unfolding primary affect-cognitive interface in preverbal (or minimally verbal) infants. It hypothesizes when behavioral and facial displays within an epoch appear to be supported by specific, primary appraisal that will need to be empirically verified. The epochs listed in the first column are well-documented periods of developmental change, widely accepted in the literature. The second and third columns list the possible expressions and valence that signal affect in each epoch. The fourth column lists the behavioral impulses to either approach or withdrawal given low to moderate arousal. The table does not cover high arousal contexts which elicit defensive or attack behaviors. Finally, the right-most column lists the appraisals in each epoch for which there is evidence based on infant capacities observed in the laboratory or in naturalistic settings. The appraisals most relevant to the first two years the Scherer (2001) model are: valence (pleasant vs. not), novelty (expected vs. not), agency (self-initiated vs. not), goal relevance (yes/no), control (responsive to me vs. not), intentional (purposeful vs. not), and power (dominate other vs. not). Because these primary appraisals depend on the infants’ growing cognitive capacities, the framework suggests when specific, primary appraisals likely support the behavioral displays of specific affect networks. Within each epoch, appraisal capacities (1) accompany functional affect to elicitors or contexts and (2) are undergirded by learning and memory processes that allow specific predictions and appraisals to be reactivated when elicitors or contexts are reinstated. Infant memories are not about isolated events, but about predictions that are retrievable in context to construct or reconstruct experience (Rovee-Collier & Giles, 2010; Sen & Gredebäck, 2021). These predictions include affect.

**Table 1:** A Working Framework Of Developing Affect-Appraisal Interface In Infancy

AGE (+ or -weeks or months)	AVAILABLE AFFECT SIGNALS TO INFANTS (face and voice)	CORE VALENCE (Negative, Positive)	CORE ACTION IMPULSE (low-moderate arousal): APPROACH-WITHDRWAL	APPRAISAL* (primary evaluative checks)
Neonate: 0–2 w	Crying, Pain scream. Endogenous smiles and reflexive smiles. All facial expressive movements are possible but not specific to situation. A cupped tongue may be unique to extreme pain screams.	N, Positivity offset when awake and sucking.	**AP**: Reflexive looking, rooting, sucking, other reflexive motor behaviors. **WD**: Crying; defensive reflexes.	None likely initially.
2 w – 12 w +/- 2 w	Crying. Non-cry vocalizations. Facial Displays: Active Interest; Knit-Brow Interest; Smiling *Common negative expressions are observable*: Anger movements are dominant and typically followed by Sad movements and/or their blends in many negative contexts. Fear and disgust components occur rarely, more commonly with high arousal.	N & P	**AP**: Same as above plus Head-turning; fixation, visual following, listening. Gross body movements: batting of arms; kicking; roll/shimmy on belly. **WD**: Head and gaze away from unpleasant stimulation.	-*Novel vs. Familiar* -early *Agency*: Self-Other discriminations: maternal preference by 12 weeks. Contingency perception (correlations of self-action with physical and social environment events) improves over time but may require 3–6 min of experience.
3–6 m +/- 1 m	Modulated crying (fuss vs. rhythmic cry; screams) Active Interest; Knit Brow Interest; Social Smiling; Smile/Laugh; Surprise; Anger, Sad, their blends; Fear configuration possible but rare. Disgust becomes specific to taste.	N & P	**AP**: All the above, plus: Reaching, grasping, banging, kicking. **WD**: Head, gaze away and body turn away	Above plus: -*Expected vs. Unexpected*: Surprise and impossible events detected. *-Goal-relevance*: APP/WD actions related to goals are repeated. *-Control and Predictability*: Rapid discrimination of noncontingency or changes in contingency. Context-dependent perceptions of control, based on recent or reactivated experiences.
7–9 m +/- 1 m	Modulated crying; rhythmic crying whining, screams. Active Interest; Knit or Wary Brow; Bi-phasic organization of Anger-Sad expressions, fewer blends; Anticipatory Smiling; Fear facial configuration remains rare; Social shyness begins.	N & P	**AP**: All the above plus manipulation, pre-crawling movements. **WD**: Throwing, dropping, pushing away what is disliked or unwanted. Gaze following is stable.	Above plus: - *Control and predictability*: Predictions are less context dependent. -*Self-Other*: Preference for familiar vs. strange adults on many measures, expectancy and predictability may underlie *Feeling Secure* (attachment). Awareness of social agents’ intentions and discrete affect. -*Goal relevance*: Increasing goal-directedness; Discrimination of goal relevant and goal irrelevant information. --Emerging *Intent*: *Wanting and Liking.*
9–12 m +/- 1 m	Same as above plus the Play face. Anticipatory smiling Head-gaze aversion combine with sober wary expressions in social situations (Shyness).	N & P	**AP**: All the above, plus early supported walking or “cruising” to explore. **WD**: Moves purposefully to and from a secure base in response to the novel, unfamiliar, or uncertain.	All the above plus: -*Intent*: Self-selected goals; gestures, pointing. -Social referencing: Shared gaze and expressions; Attends to incongruent emotion/context reactions of others to guide behavior in some contexts.
12–18 m +/- 2 m	Same as above, plus: Tantrum behaviors and “temper displays” may begin and will increase during this period.	N &P	**APP and WD**: All the above plus walking: toward and away from people or goals.	All the above plus manipulation of Intentional *Control*: saying “No”, cause and effect play). - Intent and Goal relevance: Anticipates the goals of others; Replication of another’s intentional action. -Social referencing: Emotions of others used as contextual information to manage approach & withdrawal in naturalistic contexts.
18–24 m +/- 2 m	Same as above plus: -Feelings of mastery and helplessness. -Tantrum behaviors become common in Western middle-class children. -Anger/Rage + aggression against others; Jealousy/ possessiveness. -Embarrassment (smile while looking away or alternating gaze coyly).	N & P	All the above	All the above plus: Objective self-awareness and initial self-related appraisals. Simple mastery goals.
2yrs +/- 6 m	All the above plus further development of self-conscious emotions, shame and pride	N & P	All the above.	All the above plus: *Power appraisals* may begin. Verbal concept of Happy;

*Citations for specific findings either appear directly in the text or are in review papers cited

### Birth – six weeks

#### Core affect

There is consensus in the literature that infants are born with what has been called core affect. The first aspect of core affect is its valence or positive or negative quality (Ledoux, 1989). Pleasantness or negativity characterize affect from the newborn period on. A second aspect of core affect is arousal or activation. It is described as energized, calm, agitated or lethargic body sensations based on physiological status. Valence and activation are always present and are “simple summary statements” of bodily state in the moment (Barrett, 2017; Russell, 2003). Discussion of infant core affect often omits mentioning an accepted principle of the adult nervous system, the “positivity offset” or a slight bias toward positive valence that characterizes processing of “neutral” or ambiguous events (Ito & Cacioppo, 2005). This bias means that when apparently calm or neutral, slightly positive affect processes are “idling” in the brain, presumably to allow openness to experience. This principle is an example of the close integration of valence and activation functions of core affect. The positivity offset is likely established through the action of the visual alerting network (Petersen & Posner, 2012; Posner & Rothbart, 2007). The alerting network, present at birth, helps the infant attain a calm, alert state and to detect visual and auditory information. It is reasonable to assume that infants’ resting core affect reflects the positivity offset.

Another principle of core affect accepted in adult and animal literature, but seldom discussed relative to infant affect, is the mobilization-minimization hypothesis. It states that negative affect recruits greater mobilization of physiological and cognitive resources than positive affect and therefore requires greater resources to minimize its consequences (Taylor, 1991). Therefore, differentiation of negativity will be more complex, (and presumably develop later) than positivity (Rozin & Royzman, 2001). Crying, the major negative affect signal of neonates reflects this principle. Varying in intensity and quality, crying signals primarily increased physiological arousal and nonspecific negative reactivity. Panksepp’s (1998, p.266) hypothesis that pain and distress cries might be distinguishable based on neurochemical or sound spectrum characteristics has received little support in human work. The limited study of infant cry characteristics has focused on the distinctiveness of pain, hunger, and other types of newborns’ cries and whether certain cry characteristics are useful in screening pathology (LaGasse et al., 2005; Parga et al., 2020). Crying elicits direct, effortful, and sometimes multiple caregiving interventions and there are clear individual differences in the infants’ capacity for soothability, an aspect of temperament (Rothbart et al., 2011). No studies have examined isolation- or separation-triggered distress in newborn or older infants, which Panksepp thought would specify the sad/distress system.

Facial movements signal core affect (valence and arousal) from birth. Neonatal facial displays may signal positive approach (calm interest, smiling) or negative withdrawal (varied negative-valenced displays). Seeking/interest processes function from birth if visual and auditory attention systems are intact and are the primary affect motivating infants’ initial exploration of their world. Smiling and disgust expressions are state-dependent affects in the neonate. They are triggered by taste or internal sensations even prenatally rather than by external elicitors (Ustun et al., 2022). Smiling during sleep is thought to reflect sensory pleasure or comfort and therefore positive as opposed to negative valence. Pain expressions and their latencies also vary with state of alertness and the quality and timing of the stimulus (Friedrich et al., 2017; Peters et al., 2003). Negatively valenced expressions occur during crying episodes and may display as blended or partial expressions, especially when arousal is high.

*Directional impulses.* Long described as central in behavioral/motivational accounts of emotion, action tendencies are innate behavioral dispositions to respond in a specific way to an elicitor. They include reflexes but are described more broadly as directional impulses to approach or withdraw from an elicitor (Gray, 1970; Matthews & Gilliland, 1999; Schneirla, 1959). In humans, action tendencies organize behavior and affect, either promoting and sustaining stimulus engagement, or supporting disengagement under conditions of low to moderate arousal (Watson et al., 1999). Whether approach/withdrawal is a single neural network in infants or are semi-independent is not yet clear. However, the proposed framework assumes that approach-withdrawal as well as defense neural networks operate in at least rudimentary form in the healthy newborn, constrained by infants’ limited motor and information processing capacities. Approach is the prime directional impulse of the neonate. This allows the infant brain to focus its predictive functions on the external world. Newborn approach behavior consists primarily of reflexes supporting nutritional needs and human contact. Periods of quiet alertness are limited but increase over the first days and weeks, providing approach opportunities including visual exploration of faces and tracking of objects in the immediate visual field, often while engaging in sucking which helps sustain calm, alert arousal.

All withdrawal actions of the neonate (blinking, flexor reflexes) are reflexive. As oculomotor and neck control improve, looking and turning away become possible and represent the first nonreflexive behaviors. Ideally, caregivers ensure that infants are removed from sources of discomfort and intervene in response to infant crying. They will continue to do so even as infants develop better motor and self-regulatory skills. Nevertheless, the defensive reflexes, elicited by high intensity stimulation (physical pain and discomfort), represent the early neural support of withdrawal impulses. Under these conditions, the highest level of negative valence and activation occur, including high-pitched screaming and thrashing.

*Predictions not appraisals.* Subcortical and some midbrain areas of the infant brain are developed at birth such that there may be some ascending cortical connections in the brain. Although the cognitive capacity of neonates is thought to be limited, the brain expects experience and some brain development is experience-dependent (Greenough et al., 1987). So, both forms of prediction occur, although these may not yet be considered evaluative in the sense currently suggested by appraisal theorists The visual alerting network (Petersen & Posner, 2012) likely is the basis for most experience-dependent predictions.

### Eight to twelve weeks

Once the alerting network is functional, young infants are alert for brief periods following feeding. If visual and auditory stimulation is of low to moderate intensity, primary affect and cognitive processing may occur. Theories of infant cognitive processing as well as appraisal theories of adult emotion provide some support for the launch of emerging affect-cognition links in this period. The primary affect networks described by Panksepp and colleagues suggest how approach and withdrawal affect networks incorporate core affect. Common to all mammalian brains, four affect networks (Seeking/Interest; Anger, Sad/Separation Distress, and Fear) rapidly deploy affect processing in the first year (Davis & Montag, 2019; Montag & Davis, 2018; Montag & Panksepp, 2016; Panksepp, 2011).

#### Approach affect

Seeking/Interest network function is documented in the extensive attention literature on infants’ visual exploration of the social and nonsocial environment. Interest networks must have some priority if infants are to engage the external world, to explore and generate the predictions presumed to be central to information processing. Interest affect is displayed facially during visual fixation as facial movements in the upper face that signal either receptive engagement or effortful processing (Izard, 1995). Receptive interest is the default affect if the infant is awake and alert, especially while sucking (Kessen, 1967; Korner & Beason, 1972). The face appears relaxed, eyes widened, and brows may be raised slightly. This interest expression is the modal facial display of an awake, engaged infant. In male infants, sometimes effortful interest (characterized by slightly knit brows) may be observed (Malatesta et al., 1989). Smiling may occur at the height of an “arousal jag”, allowing sustained positive affect despite a high state of positive arousal (Sroufe & Waters, 1976). Positively-valenced vocal signals may accompany approach behavior and effortful looking.

Unfortunately, interest has not been regarded as a primary affect in most infant emotion theories. Instead, the literature focuses on the fixation and duration of looking exclusively, interpreting them as a purely cognitive, rather than an integrated affect-cognitive process. While the earliest fixation behavior may be stimulus-dependent, young infants soon learn what to continue to look at and are more likely to prefer novel visual targets to familiar ones: Habituation studies demonstrate that infants learn to look away from what they have seen before. The cognitive interpretation is that they have processed the salient or relevant stimulus information and matched it to an internalized schema or prediction. They are also less interested; both interpretations suggest a basic appraisal.

By 12 weeks, alert infants can focus on targets within a specific visual field, orient to sounds, and visually track people and objects. Looking (visual fixation), maintenance of high amplitude sucking, and head-turning are actions that have been used to demonstrate that the young infants are interested in and motivated to approach neutral or positively-valenced stimuli in studies of habituation or instrumental learning. They too reflect affect as well as cognition. By the end of this period infants may contact objects in the immediate environment by gross movements of arms and legs (batting, kicking). Piagetian theory regards these and other motor behaviors as primary circular reactions, the most basic form of nonreflexive behavior. They are action impulses repeated because they are pleasurable. Smiling and cooing begin in these contexts by the end of this period.

*Withdrawal affect*. When stimuli become non-optimal, infants disengage by looking away, dozing off, or crying. Negative affect signals continue without much change. Crying remains the major withdrawal affect signal and continues to demonstrate the maximization/minimalization principle. Crying escalates quickly and involves facial movements characteristic of multiple or blended negative facial expressions: primarily anger, sad/distress, and fear. The apparent undifferentiated or unorganized infant negative expressions may be a feature of the still immature, unmyelinated withdrawal networks. Or, because sad/distress and anger neural networks share both excitatory and inhibitory influences with fear (Panksepp,^1998^), shared pathways can potentially activate multiple networks, excitatory as arousal increases, or inhibitory as arousal falls. Anger and sad display co-occurrences are widely documented in facial expression research. Sad/distress facial signals have been associated with the dampening of crying, consistent with a withdrawal action pattern (Oster, 2005).

*First appraisals.* Current neuroscience emphasizes that brain-processes create predictions about all experiences, presumably based on statistical learning or other forms of learning. These predictions continuously update through interacting midbrain and emerging cortical networks. Predictions can include information about core affect and body sensations associated with physiological changes. In Scherer’s view, affective processing begins when these predictions are integrated with primary appraisals, that is core affect, physiological changes, and impulses to approach or withdraw are linked with appraisals. Appraisal checks relevant within the first 12 weeks are valence (pleasant vs. not), novelty (Seen or heard it before? yes/no), primary agency (related to the body’s action vs. not), goal relevance (wanted yes/no). Appraisals may become more complex as infant cognitive capabilities expand, but initially, they are basic dichotomous evaluations. Evidence for appraisals comes from attention and learning studies during this period. They likely occur when salient, low to moderate intensity events are encountered and align with predictions. Memory of that correlation constitutes an affect-cognition link that will be stored as a ‘tag’ in short-term memory. Tags are hypothesized to predict the previous link associated with a particular stimulus, action, or event and will enable the salient encounter (or one like it) to be more quickly appraised subsequently. In this way, the affect-cognition link is reinstated and becomes habitual. Appraisals are not static, but continuously updated as are all infant memories (Davis et al., 2023).

Preference for novel visual stimuli and the first examples of learning using high amplitude sucking responses and head-turning (Floccia et al., 1997; Papousek & Papousek, 1983), and kicking using the mobile conjugate learning occur by 10–12 weeks (Rovee-Collier, 2013; Rovee-Collier & Barr, 2010). We cannot yet specify exactly how this learning is represented in the brain, but once we acknowledge that the brain constructs predictions about the world, and learning reflects acquired predictions, appraisals come into play. Young infants’ differential response to familiar and novel events is well established in the habituation literature. The visual preference literature, based on pair-comparison or familiarization and responses to novelty methodologies, rests on the premises that: (1) infants come to develop a prediction or expectancy based on what they have previously seen, (2) fixation of (interest in) familiar targets will decline over time, and (3) *novelty or unexpectedness* in visual information leads to the recovery of behavior. Initial attention may be reflexive but attention duration, a key habituation measure, is maintained by stimulus complexity, contextual features, and the amount of prior experience with the encountered stimulus or object. Social behavior at this age also shows that familiar and novel people are discriminated. Likewise, early learning studies use infants’ emerging approach tendencies as measures of contingency learning. Both contingent and noncontingent stimulation elicit interest from about 8 weeks but *contingency* suppresses negative affect, evidence that infants prefer responsiveness over non-responsiveness (Lewis et al., 1985; Sullivan & Lewis, 2003). Such effects replicate the effects observed for maternal contingencies during social interaction. Early and efficient detection of contingency versus noncontingency is also thought to be essential to the learning between self and other (Fotopoulou & Tsakiris, 2017; Watson, 1967). Rovee-Collier and associates’ work (2015) established that self-initiated action, objects, and their contingent relations are learned and remembered independently, supporting primary appraisals. By 12 weeks, pleasantness, novelty, and primary agency appraisals can be inferred from these studies, all of which are exclusively approach contexts. That is, experimental and naturalistic studies of infant behavior and affect utilize contexts hypothesized to be interesting or pleasant for infants thereby priming approach/interest affect by their very design.

Withdrawal responses (crying and behavior) of infants who fail to complete experimental procedures are assessed, if at all, as a “drop-out” rate due to fussiness. Whether these infants are more likely to be more distressed by novelty is seldom considered. There are few protocols for examining facial displays and behavior of infants who turn away from visual stimuli in response to a virtual looming object or the mildly stressful events of a well-baby visit. There are no studies of conditioned withdrawal so little information exists about withdrawal contexts, except that they often elicit crying and therefore presumably undifferentiated negative, nonspecific affect. One exception is pain responses which have a distinctive physiological marker of cortisol rise in addition to intense crying and pain facial cues (Lewis & Ramsay, 1995).

### Three to six months

*Approach affects: Interest and Anger.* Until this epoch, approach affect has consisted primarily of seeking/interest but infants tolerate greater levels of positive arousal such that enjoyment smiles and laughter become more frequent, peaking at 12–14 weeks. Learned behavior now constitutes a secondary circular reaction, a response focused on the external world and repeated because of a positive consequence. Infants are interested in objects as well as people and will soon be reaching for them. Appraisals of goal relevance and control are now possible. Goal relevance in this epoch is an evaluation of wanting: Do I want it? Yes/no. Control is an evaluation of contingency: Is responsive to me? Yes/no (Sullivan & Lewis, 2003). Although contingency perception occurs in social interaction prior to 12 weeks, laboratory studies of contingency learning with objects have been able to directly manipulate contingent and noncontingent information to study its impact on behavior, affect signals, and physiology (Sullivan & Lewis, 2003; Sullivan, 2016). Once infants have learned to produce a contingent event, actions to regain it are evidence of goal-relevant behavior. Negative affect signals, primarily anger/approach, affect typically occur when goal-relevant action is thwarted. This type of study provides one way to study anger/approach affect relative to withdrawal affects, especially sad facial signals which occur at low levels.

Anger-approach networks reflect annoyance, as well as “getting mad”. Attentional focus narrows and is no longer exploratory but is directly focused on regaining the goal of approach (Gable & Harmon-Jones, 2010). Behavior is energized to push through or remove any obstacle or irritation. Effort increases and actions may become more forceful but regaining a wanted goal is key. Infants displaying interest typically display little or no angry affect until access to the wanted object is blocked as hypothesized in the Panksepp model. When the goal access is restored, anger expressions diminish and interest returns (Lewis et al., 1992). Panksepp thought mammalian anger occurred primarily in response to restraint by a predator, but blocked access to food and other survival-relevant goals were also important. Blockage of wanted goals may be the more important aspect for infants. Although restraint has been studied in infancy, it does not reliably elicit infant anger expressions and action tendencies in young infants. Restraint via infant swaddling, both in nontraditional cultures and in laboratory studies, lowers rather than increases arousal (Brackbill, 1971; Dixley & Ball, 2022). Arm-restraint does elicit anger facial signals and struggling in 4- to 5-month-olds but it is not the initial or modal expression (Bennett et al., 2005; Sternberg & Campos, 1990). Instead, interest expressions frequently occur at both 4- and 12-months, suggesting that infants may respond initially to restraint as either a novel event or a play situation (Liu et al., 2022). Anger expressions to restraint significantly increase only after infants have become independently mobile when this context may be more likely perceived by infants as blockage of wanted action (Bennett et al., 2005; Stenberg et al., 1983).

Blocked access to objects in which infants previously displayed interest are commonly described as frustration paradigms. After 8 months, anger/approach observed in such paradigms has been termed “frustration”, “frustrated approach” or “frustrative non-reward”. Consistent with the model offered here, the affect elicited is anger/approach. Anger/approach, marked by pre-specified, objectively scored facial actions and increased behavior directed to regaining a goal of interest occur consistently and reliably when access to that goal is blocked *prior to 6 months* (Crossman et al., 2009; Sullivan, 2018). Learning/frustration paradigms allow infants to actively demonstrate approach behavior and interest during the study’s learning phase. Subsequently, an extinction phase or other disruptions of goal access allow observation of the occurrence, extent, and quality and quantity of anger/approach and any withdrawal affect as a function of prior learning, interest affect, and a resumption of interest in the goal when it is reattained (Lewis et al., 1992).

*Early withdrawal affect: Sad distress and blends*. Panksepp (1998) viewed sad/distress as a major network of mammalian social-emotional processes and the basis for distress calls. He also viewed this network as the source of loneliness, sorrow, loss, and grief. Sad distress dampens approach and directs attention away from a goal, fostering a broader focus of attention; there is cessation of motor action and giving up previous activity (Gable & Harmon-Jones, 2010; Sauce et al., 2018). Cries for help and comfort-seeking are the action impulses of this affect. Panksepp also argued that separation distress and specific attachments emerged simultaneously with the maturating of motor systems that allowed the young to wander away. Sad/distress is clearly relevant to infants’ attachment behavior and being able to seek help from others (Sroufe et al., 1983). However, sad/distress occurs outside of attachment and social settings in infants and children.

In nonsocial contexts, sad/distress may signal any perceived loss of a wanted object. Sad/distress facial signals are evident in 4- to 6-month-olds when noncontingent and partially contingent events follow a period of contingency (Sullivan & Lewis, 2003; Sullivan, 2018). In these less responsive contexts, sad displays and dampened activity may signal disappointed expectancy or helpless distress. They imply only that the affect processes for distress can activate when a pleasant, wanted goal is perceived as lost and not recoverable, a finding observed in animals as well as older children (Maier & Seligman, 2016; Sauce et al., 2018).

Consistent with Panksepp’s findings of shared excitatory/inhibitory links between anger and sad networks, infants’ facial displays of sad/distress often co-occur with anger signals or are blended with them from 4 months on (Camras & Shutter, 2010; Sullivan, 2018). In older infants and toddlers, tantrums are comprised of both anger and sad affect in a biphasic, organized process (Potegal & Davidson, 2003). There have been few studies of anger-sad processes between 4 and 12 months in response to frustrated nonreward, so it is unclear when this bi-phasic organization first emerges and there is no evidence that anger and sadness/distress are opponent processes (Sullivan, 2018; Zhan et al., 2018).

Blending of sad facial movements with those of anger in a single expression is often cited as evidence of undifferentiated negative affect. However, hypothesized interactions between anger and sad/distress networks suggest another possibility: Blending or co-occurrence of sad/distress with other affect processes in the same context may likely occur throughout infancy. Differentiation may require experience in context and specific appraisals related to lack of control or loss. For example, the amount of blended anger/sad displays decreased in response to contingency disruption with a second disruption experience (Crossman et al., 2009). Sad/distress displays also decreased over time but anger/approach did not (Crossman et al., 2009; Sullivan, 2018). Other potential explanations for blends or co-occurrence of the two affects can be hypothesized other than that they are undifferentiated signals. They might reflect either downregulation of anger or conflict between approach-withdrawal networks. The needed, sequential data on the patterning of these affect signals over time have not been reported, so the conclusion that their blending and co-occurrence reflects undifferentiation is premature.

*Withdrawal affect as arrested approach*. Proposing innate affect processes for fear raises the objection that behavioral signals of fear are not reliably observed in contexts designed to elicit them throughout most of the first year; for example, in response to the approach of a novel, nonsmiling, masked stranger; the sudden, loud popup of a jack-in-the-box; growling and intrusive toys; visual cliffs etc. (Bennett et al., 2005; Camras & Shutter, 2010). Such findings are usually explained by either proposing that infants lack the cognitive skills and life experiences to experience fear, or by assuming undifferentiated negative affect. Yet, the initiation of fear networks can be observed at 3–6 months consistent with Panksepp’s findings on the inhibiting effects of fear networks on seeking/interest. Panksepp (1998), proposed fear neural networks shared excitatory and inhibitory paths with interest. He thought fear networks functioned primarily to minimize threat of bodily harm, as opposed to energizing flight or retreat. Fear networks include both low arousal affects such as anxiety, uncertainty, and foreboding as well as high arousal affect such as terror. Through reciprocal interactions with interest/seeking and sad/distress networks, stilling or freezing responses and hypervigilance serve to inhibit approach affect and were examples of fear affect in his work. Behavioral freezing and gaze aversion have been reliably observed in infants to other types of expectancy violation at 5 and 7 months and have been interpreted as detection of the unexpected as well as uncertainty (Scherer et al., 2004). Low arousal fear qualifies as withdrawal affect because it halts approach and promotes retreat or avoidance of something unwanted. When young infants lack the motor skills to escape or flee from threat, stilling (the abrupt cessation of ongoing behavior), facial sobering, or turning away may be the earliest manifestations of low-arousal fear affect. Tension, if observed, may occur in knit brows or lower eyelids, facial movements sometimes described as wary interest. All these behaviors are within infants’ repertoire before six months. Thus, low arousal fear behaviors and facial components might be most likely when (1) there is actual physical trauma or pain (pediatric inoculation is one naturalistic context that could be explored ethically), and (2) where an expectation is established and abruptly and strongly violated.

The second context is like those employed to elicit surprise displays. The facial and behavioral signals of surprise and fear are similar. Fear facial displays feature more extreme eye widening, and tension evident in raised, straightened brows and the lower eyelid/cheek area. Surprise is generally regarded a “neutral” or mildly positive facial expression observable in naturalistic contexts after 4–5 months of age and is judged by rapid brow raises that dampen quickly to interest (Izard, 1995; Sullivan & Lewis, 1989). Surprise-eliciting contexts elicit fear at 4 and 12 months of age in about 12% of infants. Fear expression is also the third most frequently observed affect at 4 months in response both surprise and masked stranger eliciting contexts (Bennett et al., 2002, 2005). Both fear and surprise expressions are observed during opening minutes of contingency training as infants learn to tug a ribbon to activate a slide/sound show. These displays occur prior to the time that the contingency is perceived, but individuals differ in these displays (Sullivan & Lewis, 1988). Do fear and surprise displays reflect the same affect processes but lower levels of arousal in some infants? Further work is needed but it appears that low arousal fear networks may activate when some infants encounter expectancy violations and when contingencies are either unpredictable or uncertain. The degree to which temperament differences, sensory thresholds, or prior experience predict whether surprise or fear will be displayed is unknown.

*Infant memory and appraisals*. Infants’ memory of learned information underlies the rapid expansion of everyday predictions about people, objects, and their relation to one another and to infant actions. Infants are active competent learners of the world’s predictive framework. Effectively, they are “inventory control officers”, learning “who goes with what and when” consistent with developments in the hippocampal functions as a general-purpose coder of content limited or ordinal structures (Buzsáki, 2019; Lisman et al., 2017; Rovee-Collier, 1996). Consequently, during this epoch infants form individualized, increasingly elaborate databases for retrieval of predictions and their appraisal “tags” that can be maintained for weeks given adequate experience and reactivations (Rovee-Collier & Barr, 2010; Rovee-Collier & Giles, 2010). Infants’ predictions are still limited and based primarily on their approach behavior but appraisals may now include whether events are expected or controllable (responds to my action? Yes/No). Learning and memory for any specific infant experience is dependent on context, including incidental background features. When predictions are reinstated through repetition, additional features may be incorporated into the prediction, an effect known as post-event bonding (Rovee-Collier & Barr, 2010; Rovee-Collier & Giles, 2010). Experiences generalize only as contextual details are forgotten or when sufficient contextual variation promotes it (Rovee-Collier, 2013). The updating of memories suggests how appraisals may be updated and incorporated into predictions. Affect experienced during learning itself impacts memory and capacity for relearning at this age (Crossman et al., 2009; Lewis et al., 1992; Singer & Fagen, 1992). Affect during learning or its immediate aftermath may function as a contextual cue or setting event linked to remembered predictions and appraisals. Negative affect can interfere with the predictions underlying retention and reactivation. The above-cited work concerns appraisals of approach-affect since it is derived from studies of reward-learning. Retention of predictions and appraisals for withdrawal contexts have not been studied but they are theoretically possible given this framework.

Infants’ capacity to perceive and appraise self-initiated events advances beyond simple expectancy violation during this period. Differential responses of infants to contingent and noncontingent information with only brief experience demonstrates that after 12 weeks predictable and controllable events are rapidly perceived in social and nonsocial contexts (Sullivan & Lewis, 2003; Weinberg & Tronick, 1996). Sensitivity to noncontingency demonstrates that infants perceive no correlation between their own actions and an event. Infants will act to regain only contingent (controllable) or partially contingent (predictable, but less controllable), but not noncontingent events. This is developmental change: At 2 months, infants attend to and are interested in and aroused equally by noncontingent and contingent information for several minutes. By 4 months, contingency exposure leads to increased responding and interest within the 4–6 min, while exposure to noncontingency rapidly leads to negative affect and subject loss due to crying (Lewis et al., 1985). Affect displays at 4–6 months also confirm agency appraisals. Contingency disruption, created by removing access to the expected “goal”, leads to predominantly anger expressions and increased activation, called an “extinction burst” (Lewis et al., 1990; Sullivan et al., 1992). The extinction burst marks perception of a correlation between self-initiated action and a goal since this phenomenon is not replicated in affect-less robotic simulations of infant learning (Zaadnoordijk et al., 2018). Although not full cause and effect understanding, the extinction burst reflects appraisal of self-initiated (primary agency) and goal-relevant action.

When infants are shifted from contingency to partial contingency (in contrast to extinction), they increase their goal-directed action, but their negative affect, including anger, sad, and blended component displays, is also greater (Crossman et al., 2009; Sullivan & Lewis, 2003). This means that infants still perceive that the partial contingency is related to their actions, but dislike or experience distress at having to work harder to control the goal.

When infants with contingency experience are shifted to noncontingency, their responding decreases dramatically (in contrast to extinction), and sad displays increase in the negative affect mix (Sullivan & Lewis, 2003; Sullivan, 2018). Therefore, infants perceive that they no longer control the goal and this loss is marked by distinctive affect displays.

Sad/distress expressions and response decreases during a noncontingency shift parallel reports of withdrawal observed in classic studies of learned helplessness in animals and children (Maier & Seligman, 2016). Assessment of appraisals of contingent vs. noncontingent information in infants have not been fully explored since most of this data is based on initial responses following a single, brief learning experience. Effects of repeated exposure to noncontingency or the interjection of intermittent noncontingency is unknown.

By six to seven months, infants can detect novel versus familiar and expected vs. unexpected events. They detect predictable external events and their correlation with self-initiated actions. These behaviors are consistent with a primary appraisal constellation hypothesized by Scherer and can be thought of as “the four U’s“ (unfamiliar, unexpected, unpredictable, and uncontrollable).These appraisals are hypothesized to occur whenever infants encounter uncertainty in their interactions with people and objects.

### Seven to nine months

*Continued maturation of withdrawal affect*. Until this point, approach behaviors were limited to visual following, kicking, batting, reaching for, or leaning toward objects. A seated posture, free movement of upper limbs, pre-crawling movements, and standing with or without support now allow the infant new perspectives on its body and the means to approach objects (Adolph & Hoch, 2019). Play experiences and emotional contagion during interactions with caregivers from this age through late infancy continually reactivate smiles of enjoyment and laughter, “Happy” affect experiences that will eventually become the first categorical emotion label acquired by Western children (Denham, 1998). Surprise reactions, observed after 4 months in laboratory settings, occur naturally during social play (Parrott & Gleitman, 1989). Withdrawal affect is more readily evident in social contexts. Wary and shy behaviors may be observed in response to strangers (Bohlin & Hagekull, 1993).

Early manifestations of fear neural networks have been called “wariness”. It appears in the third quarter of the first year as “stranger wariness” and “secure base” behavior in the attachment literature, “white coat syndrome” in the pediatric literature, and as behavioral inhibition in the third of infants who regularly display it as an aspect of temperament (Fox et al., 2021). A hierarchical neural organization of fear processes reflects infants’ physical distance from threat is consistent with Panksepp’s view (LoBue, 2014). More distal threat promotes cautionary behavior but freezing occurs in response to more immediate threat. Some investigators have resisted labeling wary behaviors as “fear” because few fear/terror expressions occur even when infants cry or retreat to the caregiver (Camras, 2022). Yet, wariness demonstrates that activation of low arousal fear processes occurs in conjunction with the maturation of the prefrontal cortex at this age.

When stimuli are highly arousing, aversive, and potentially intrusive or threatening, retreat impulses should override approach tendencies with rapid “emergency” physiological activation supporting escape or flight attempts (LoBue, 2014). Flight rarely occurs in the laboratory contexts used to study infant fear. Instead, retreating to “secure base” marks infants’ new ability to use the caregiver to buffer low arousal fear or uncertainty. Extreme fear responses may thus fail to occur because (1) the fear-eliciting stimuli used may not signal impending harm, or (2) infants typically are tested in the caregiver’s buffering presence. The contexts typically used to elicit fear (visual cliff, masked stranger, or strange, noisy toy) may never rise above the emergency threshold to fully engage fear/flight activation. Instead, they elicit approach-withdrawal actions and affect only, except for those infants who are temperamentally vulnerable or sensitive to perceived threat (Buss et al., 2013; Fox et al., 2021; LoBue, 2014). Another factor limiting “emergency” flight at this age is that the infant still lacks the cognitive skills and experience to anticipate danger without prior direct experience with a dangerous elicitor. Other than turning away, flight responses also require efficient late-maturing locomotor responses. Instead, withdrawal tendencies and sad/distress affect both signal a need for help from caregivers. Without an imminent threat, the emergency response system may never be fully challenged.

*Appraisals.* Infants can now directly explore their agency and control of objects in the physical world by dropping, throwing, and other forms of manipulation and they enjoy cause and effect play in many forms. Consequently, appraisals of agency and control become less context dependent (Rovee-Collier & Barr, 2010). Predictability and control remain important features of infant behavior in play contexts as infants encounter ambiguous or highly novel objects or people (Parritz et al., 1992). Goal-directed behavior becomes intentional and infants may predict other’s intentions (Woodward et al., 2009). Infants want things and communicate their wants and likes in basic ways. Daily reactivations of wants and likes become the basis of infants’ affective experiences of pleasure and contentment. Blockage of their likes and wants produces increased efforts to regain them and anger/approach. Behaviors such as hitting, or biting, may appear at this time (Lorber, Del Vecchio, & Slep, 2015). They demonstrate that self-initiated aggressive behaviors occur as instrumental aggression when infants’ goals are blocked. Uncontrollable loss elicits sad/distress affect. Infants discriminate some adult expressions in context at this age, if not earlier (Montague & Walker-Andrews, 2001; Walker-Andrews, 1997) and may use caregiver expressions to guide their own action in unpredictable, incongruent, or uncertain contexts, examples of social referencing (Reschke et al., 2017).^2^

**Fig. 1:**
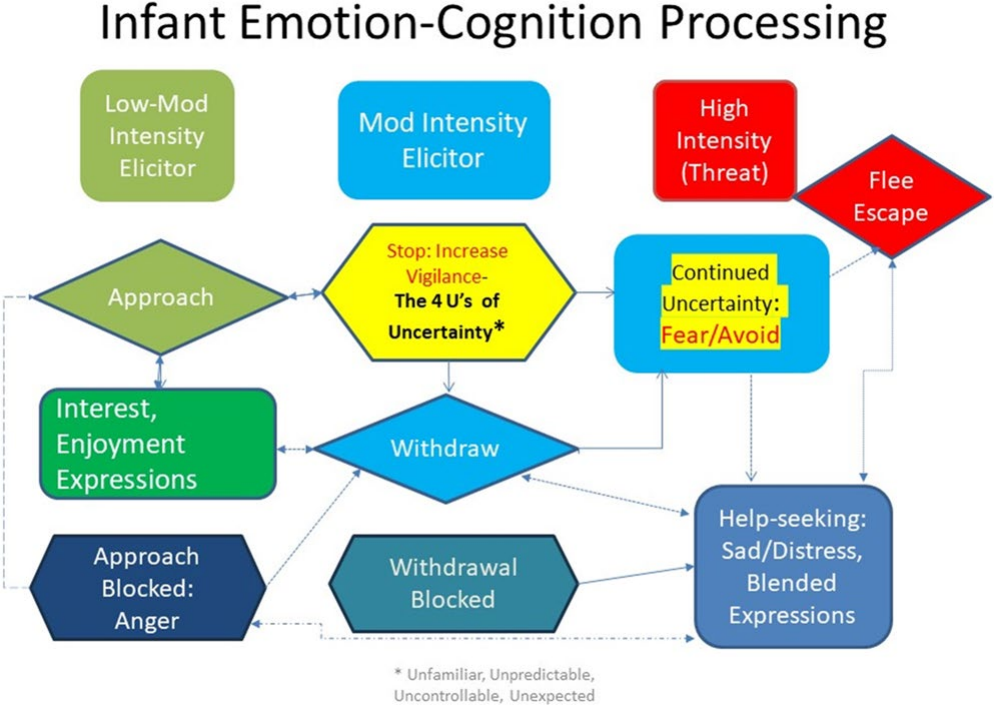
A Proposed Infant Affect-Cognition Interface. This schematic summarizes a proposed affect-cognition interface based on the integration of affect network and appraisal models. The top row divides salient events into three arousal intensities: low to moderate, moderate, and high. Infants’ perception of intensity is individually variable. Approach affects (green) occur in response to low or moderate intensity events and require little or no appraisal for resulting interest and enjoyment. If approach affect is blocked, anger/approach may result. However, withdrawal and sad/distress may also occur (light blue boxes and dashed arrows) depending on differences in infant temperament, experience, or contextual features. With moderate intensity events, approach affect may be inhibited for further processing (yellow), initiating one of the four appraisals of uncertain or ambiguous events. If appraisal results in an evaluation of any uncertainty, withdrawal and sad/distress result (dashed, bidirectional arrows). In response to continued uncertainty, avoidance and help-seeking become likely. The dashed arrows show hypothesized interactions between sad/distress and fear networks (yellow with red). High intensity elicitors (red) are proposed to result in immediate flight or escape. Flight or escape may also flow from continued or prolonged uncertainty of less intense elicitors

A functional interface between approach/withdrawal affect and cognition established by the end of this period, is summarized in Fig. 1. It diagrams the activation of affect neural networks in response to elicitors of low to moderate, moderate, and high intensity and links to primary appraisals. The hypothesized processes do not require categorical emotion constructs on the part of the infant, nor differentiation of discrete states. For low to moderate intensity events, approach is the default affect leading to exploration, interest, and enjoyment in most contexts. Infants will withdraw as positive affect wanes without significant negative affect if they can re-engage interest elsewhere. If approach affect is blocked, anger and the affect experience of wanting results. Ensuing appraisals and actions determine whether infants overcome the blockage or give up and withdraw. Temperamentally soothable infants will withdraw and re-engage interest elsewhere. Otherwise, infants may show sad/distress as anger processes downregulate, or if they perceive that a wanted object or person is not recoverable. Among temperamentally irritable or anxious infants, sad/distress processes may function to signal need for caregiver support.

What infants perceive as moderate intensity events or contexts will vary. If arousal exceeds their “moderate” threshold, fear network processes engage. Approach stops and increased vigilance (wary attention) allows processing time for the four primary appraisals (the Four U’s: unexpected, unfamiliar, unpredictable, uncontrollable). Infants may continue approach or withdraw from the context with minimal level of negative affect based on their appraisal. Withdrawal affect signals not wanting contact or engagement. Continued uncertainty at any point will lead to increased distress, primarily sad/distress but also potentially fear or anger. Appraisals determine the flow of negative affect processes. Sad/distress networks’ shared excitatory influences with anger and fear networks and ongoing appraisal may allow for their activation. Blocked withdrawal or a high level of perceived threat are hypothesized to directly elicit flight or escape responses.

### Nine to twelve months

Approach and withdrawal affects are reflected in new behaviors in this epoch but the basic interface between affect and cognition remains. Primary appraisals continue and expanding cognitive skills, including social cognition, allow for evaluations of intent. Infant goal-directed behavior itself is increasingly intentional. Signs of intent include shared gaze with interactive partners, imitation, and pointing. Infant goals are not pre- planned, but infants know what they want to have. Independent locomotion alters infants’ perception and cognitive capabilities (Adolph & Hoch, 2019; Campos et al., 2000). Rovee-Collier (1996) described infants at this age as “proficient” mapmakers. Predictions about “who goes with what” now include “where”. Self-other distinctions are clear and reflected in social settings and by gesture. Attachment to a caregiver provides infants with a secure base from which to wander and purposefully explore. At the same time, the context specificity of memory for predictions declines further (Learmonth, Curevas, & Rovee-Collier, 2015; Rovee-Collier & Barr, 2010), suggesting that infants can now generalize experiences of predictability and control to new contexts and social encounters.

Studies have not attempted to determine whether violations of predictability vs. control produce differences in the quality of negative affect at this age, however appraisals related to loss of predictable or contingent, pleasant events should continue to result in sad/distress behaviors while blocked access to “wants” and “likes” continue to result in anger/approach. Restraint and limit-setting may now elicit anger expressions, especially when self-selected goals are blocked or thwarted by objects or people (Lewis et al., 2015). Aggressive behaviors (hitting, biting, pushing) are still infrequent and associated with maternal reports of high anger reactivity, suggesting a lower threshold for frustrative non-reward behavior and affect (Lorber, Del Vecchio, & Slep, 2014, 2015). Analysis of the dynamic unfolding of anger expression and its regulation suggests that aggressive behaviors in toddlers follow unsuccessful toy-recovery attempts (blockage) and failed bids for help (nonresponsive caregivers) (Liu et al., 2022).

### Twelve to eighteen months

Appraisals of personal intent and control are hypothesized based on observations of infants’ mastery attempts, their testing of control in interactions with parents through the word “No”, and their increasingly willful behavior. Caregiver limit-setting and infants limited capacity to achieve their likes and wants independently are expected to make anger-approach common and its regulation a socialization priority at this age. Tantrum-like behaviors begin by the first birthday in many toddlers and increase during this period, as do less intense forms of anger with mothers reporting that their infants have “a temper” or “get mad” daily (Sullivan & Lewis, 2012). Such behavior may escalate into tantrums.

Tantrums in young children are complex, temporally organized, intense reactions composed of bi-phasic anger and sad behavioral components, in which the sad components are associated with downregulation of anger (Potegal & Davidson, 2003). The presence of sad/withdrawal responses following initial anger response within a tantrum signals that external support for regulation is needed. Liu et al. (2022) found that increased anger was associated with lower support seeking and with unresponsive parents. Moreover, when support for emotion regulation was unmet, escalation of anger was associated with aggressive behavior only in the following situations: during attempts to escape restraint, as predicted in Panksepp’s model (Liu et al., 2022), and when there was anger/annoyance on the part of the caregiver (contagion), interparental conflict (modeling), or low parental responsivity (contingency appraisal) (Lorber, Del Vecchio, & Slep, 2015; Sullivan & Carmody, 2018). The escalation of anger/approach into rage/fight thus required additional factors and a different appraisal than goal recovery. Lewis (1993) and others also suggested that rage/fight differs from anger/approach. Once infants understand their wants are being directly blocked or threatened by another and have a sense of self awareness, rage/fight responses may be activated when infants perceive direct blockage of or threats to their wants by another.

### Eighteen to twenty-four months and beyond

Increased language and the growth of social cognition drive infants social understanding of other’s emotions as demonstrated in the social cognition literature. However, our focus remains on appraisal processes of infant’s own affect experience. During this period, most of the primary appraisals described by Scherer and Moors (2019) are theoretically possible. The last emerging is the power appraisal. Power appraisals are related to the autonomy struggles of the “terrible twos” and the toddler’s attainment of an objective sense of self by 18–24 months of age (Lewis, 1993). Differences in anger/approach predict persistence and mastery at this age, not power assertion (Lewis et al., 2015; Sullivan & Lewis, 2012). Power appraisals are related to deliberate, willful aggression initiated towards another centered on the control of objects or persons. Aggression and rage/fight, evidence of power appraisals, may be observed in triadic contexts where the child wants to assert or reassert control or dominance. It may appear as jealousy or the intent to retain or wrest possession of objects or persons from another (Masciuch & Kienapple, 1993).

The attainment of objective self-awareness and the involvement of self in appraisals arises by age 2 (Lewis, 2007). One marker of self-awareness, besides mirror self-recognition, is me/mine pronouns which toddlers begin to use at this age indiscriminately for anything wanted. This attainment of self-awareness, while not an appraisal, makes self-awareness of the infant’s own affect more likely and therefore supports higher order processing of affect and the differentiation and the first emotion categories. It also gives rise to an entirely class of emotions during the preschool period. It transforms emotional life because it allows children to begin evaluating themselves and their behavior against some external standard or rule. The self-conscious emotions include shame and pride (Lewis, 2013). Infancy ends at 2 years with the potential for these more complex, self-conscious emotions and for more elaborated self-appraisals (Leary, 2007; Lewis & Sullivan, 2005).

## Generalizability of the framework

The framework presented relies on studies conducted predominantly with middle-class infants and primary female caregivers. Despite this limitation, it should generalize across cultures due to the canalization of early sensorimotor development. Learning experiences and differences in social and physical environments within and across culture may produce variability in age of emergence of the interface, but absent major traumatic perturbations, the developmental sequence will be the same. Testing this challenging hypothesis would involve at minimum independent assessment of appraisal capacities, followed by observation of related, specific affects. For example, response to expectancy violation in a low-affect context could be used to divide young infants who have capacity to detect a violation from those who do not. Subsequent comparisons of these groups’ affect, behavior, and physiological responses to expectancy violations eliciting interest/surprise or enjoyment to a novel event or anger to contingency blockage then could be made across culture.

*Temperament differences.* In outlining the framework, we noted when aspects of temperament that might influence variability of the interface within developmental periods. Temperament describes variation in infants’ positive and negative emotionality, motor reactivity, soothability, and attentional orienting and its effortful control and is thought to reflect genetic variability in reactivity and arousal regulation. Temperament differences are not themselves affect processes but biophysiological differences underlying the threshold, intensity, and dampening of affect processing. Positive temperament and specific forms of negative emotionality (distress to limits/frustration-proneness and distress to novelty/anxious, inhibited temperament), map directly onto three of Panksepp’s affect networks: interest, anger, and fear. They can be regarded as differences in the threshold and processing speed within these affect networks but they should not alter the sequence of the interface. For example: inhibited infants may show distress to novelty more readily or at a younger age than infants who are not inhibited because their biophysiology facilitates the emergence of the interface given correlated affect-cognitive processes. Active and soothable (more rapid down-regulation) temperaments may vary in the intensity or dampening of affect across affect networks generally, since they are primary forms of regulation; i.e. soothable infants may more readily dampen positive as well as negative affect. The neural mechanisms of any such effects would need to be identified.

## Summary and future directions

This developmental framework was prompted by dissatisfaction with the theoretical view that emotion experience in infants requires verbal or nonverbal categorization capacities. This exclusively conceptual view suggests that for most of the first year of life, affect is minimally processed, if at all, and therefore cannot interface with co-emerging appraisal, learning, or memory processes in younger infants. It implies (although Barrett and colleagues may not directly say this) that affect is not encoded in infants’ rapidly developing brain networks and that early affect processes do not influence experience directly or indirectly in infants’ daily interactions with the social and physical world. These assumptions fly in the face of ongoing research about the impact of early experiences on brain networks, infant attention, learning, and memory research, and well-demonstrated findings about the importance of early social-emotional interactions. We propose early integration of cognition and affect networks without abandoning the idea that higher order cognitive capacities, such as attainment of categorization ability and attainment of objective self-concept change emotion life significantly and we have built them into the framework. Our hypothesis is that prior to their development, affect-cognition processing still occurs via appraisals and underlies infants’ life experience.

Relegating affect experience to the emergence of conceptual abilities after 9 months ignores considerable evidence that affect processes and affect-cognition links function much earlier. This developmental overview proposes that specific affect-cognition links emerge continuously during infancy. It advocates integrating affect network and appraisal theories of emotion into a unified developmental framework describing how early affect cognition links emerge. This framework assumes that affect accompanies all self-relevant infant-environment transactions but that the experiences infants have, and the actions of which they are capable, are constrained at every age by their motor development, temperament, and their appraisal capacities. The framework asserts that the emergence of higher-order processing in a developing brain is part of the ongoing integration of affect and cognition that begins with basic appraisals about the qualitative features of people and objects that infants encounter and their reactions to them. It allows for generalized, simple categorical affect concepts, such as happy, mad, and sad to emerge with expanded infant cognitive capacities as affect is further processed. Continually reactivated approach processes allow the pre-conceptual pleasant experiences, needing, wanting, and liking affect experiences. Withdrawal processes with their more complex neural organization depend specifically on appraisals of predictability, control, intention, and ultimately power.

Hopefully, the framework also provides a scaffold within which hypotheses and empirical work, focused on infant affect and primary appraisals, can be designed especially within the first year of life. The nature and methodology of such studies will necessarily vary depending on the specific affect-cognition link or processes being examined. Memory for predictable and controllable or noncontrollable events, and how affect influences them would seem to be an important area to begin study. For example, affect-appraisal tags might be studied by examining whether pre-exposure to positive affect or mildly negative affect influences learning, the affect experienced during shifts in predictability and control at various ages, contingency memory, or its reactivation. A research program also might be designed around the age and circumstances under which withdrawal affect can or cannot be conditioned. Whatever paradigms emerge, it is hoped this framework will encourage researchers to move away from questions such as when anger or fear “first” emerge, or when a particular facial expression configuration is “really” a specific, discrete emotion. Instead, the research should address how affect signals relate to specific appraisals, whether multiple or shifting appraisals are possible, as well whether as individual differences in affect processing are related to temperament and infants’ previous life experience.

*FOOTNOTES*

1. MAX (the Maximally Discriminative Facial Coding System, Izard, 1995) is associated with Differential Emotion Theory (DET, Izard, 1995) but is not widely used to score infant facial affect because its original theoretical underpinnings were discredited. MAX remains a valid and reliable coding tool for infant expressions and is relatively easy to learn. The two facial coding methods available in infancy (MAX and Baby FACS; Oster & Rosenstein, 1993) score the same movements although Baby FACS is more granular and focuses on more individual facial actions rather than specific sets of correlated movements.
2. The age when infants first can differentiate adult emotion displays is still unsettled and appears to vary by whether multimodal or unimodal cues are provided, culture, the specific emotion context, as well as other factors. The age noted here is conservative since 7 months has been more reliably reported for visual recognition. Earlier and affect specific findings have not consistently replicated (Ruba & Repacholi, 2019). It is unclear whether they reflect only discrimination of positive versus negative emotions.

## Data Availability

This article did not analyze any new datasets but presents and overview of a body of published empirical data for which complete citations are provided in the reference section. This work proceeds from within an ongoing theoretical discussion about the organization and interpretation of emotion in human infants. This article is an overview and discussion of theoretical interpretations of previously published, publicly accessible studies. Therefore, ethical review was not required. Likewise, since no human subjects were enrolled, there was no need for an informed consent process. The corresponding editor reports that none of the authors have any conflicts of interest.
